# Hostility, Physical Aggression and Trait Anger as Predictors for Suicidal Behavior in Chinese Adolescents: A School-Based Study

**DOI:** 10.1371/journal.pone.0031044

**Published:** 2012-02-16

**Authors:** Ping Zhang, Robert E. Roberts, Zhuoya Liu, Xian Meng, Jie Tang, Lin Sun, Yizhen Yu

**Affiliations:** 1 The Department of Child, Adolescence and Woman Health Care, School of Public Health, Tongji Medical College, Huazhong University of Science and Technology, Wuhan, Peoples Republic of China; 2 Division of Health Promotion and Behavioral Sciences, School of Public Health, The University of Texas Health Science Center, Houston, Texas, United States of America; Catholic University of Sacred Heart of Rome, Italy

## Abstract

**Purpose:**

This study explored the extent to which trait aggression is associated with suicidal behavior in a nationwide school-based sample of adolescents.

**Methods:**

A nationwide sample of 14,537 high school students in urban areas of China was recruited. Information concerning suicide ideation, plans, attempts, trait aggression and other risk factors was collected by a self-reported questionnaire. Multivariate regression analyses were employed to predict suicidal behavior.

**Results:**

Approximately 18.5% of students reported suicide ideation, 8.7% reported suicide plans, and 4.1% reported attempts during the past one year. Hostility and trait anger had a significant positive association with suicidal ideation. Hostility and physical aggression were positively related to suicide plans. Hostility had a positive correlation with suicide attempts, while trait anger was inversely associated with suicide attempts.

**Conclusions:**

This study suggests that hostility, physical aggression and trait anger may be able to be used to predict suicidal behavior among adolescents. Suicide prevention programs should target at attenuating the severity of hostility, anger and physical aggression. But teachers and parents should also give close attention to students with low trait anger.

## Introduction

Suicide and suicidal behavior are serious social and public problems in China and around the world, particularly in children and adolescents [1]. According to the 2004 World Health Organization (WHO) Mortality Database, the suicide rate of youth aged 15–19 in China is 4.0 per 100,000 with a gender ratio of 0.7∶1 (males∶ females), which is a little lower than that in Japan (6.4 in total, a male to female ratio of 2.3∶1) where suicide numbers are as many as those in China [2]. As to children aged 5–14, their suicide rate increased from 0.7 to 0.9 per 100,000 for males and remained 0.8 per 100,000 for females in the 1990s, much higher than that in Japan (0.5 for males and 0.3 for females) [3], [4]. Previous studies have also showed that suicide rate increased at the late teens and continued to rise until the early twenties [5]. In addition, the prevalence of nonfatal suicidal behavior, including suicide thoughts, suicide plans, deliberate self-harm, and attempted suicide, are also common in 15–24 year olds adolescents [6]. In the United States, the average rate of adolescents reporting suicide attempts in the past one year is 6.4%, 12.4% reporting suicide plans, and 19.3% reporting suicide thoughts [6]. In rural areas of China, the prevalence of suicide ideation, plans and attempts among adolescents are 19.3%, 10.5%, and 7.0%, respectively [7]. Although only a small proportion of suicide attempters complete suicide eventually, suicide attempts are significant predictors and indicators of subsequent completed suicide [8]. As a result, understanding youth suicide and suicide behavior and finding useful prevention strategies are extremely urgent.

One of the most concerned risk factors for suicide and suicidal behavior is aggression/violence. First of all, they have a common basis in pathophysiology, the abnormal serotonergic system. For example, lower level of cerebrospinal fluid 5-hydroxyindolacetic acid is not only associated with the increased risk of future suicide among adolescents, but also with the severity of lifetime aggression [9], [10] On the other hand, psychologically speaking, aggression is an important diathesis part of suicide behavior according to the stress-diathesis model proposed by John Mann [11]. Individuals with this diathesis might be likely to experience more suicidal feelings and thoughts, and to be more impulsive. He also hypothesized that the risk for suicidal behavior was determined both by a psychiatric illness and by the diathesis, and the diathesis or trait-like predisposition was more important than the severity of the illness in predicting suicidal behavior.

Previous studies in both high-risk individuals and school-based populations have found that trait aggression may act as a predictor of future suicide and elevate the risk for suicidal behavior. For example, Keilp JG et al's study found that it was aggressiveness that held the most importance in predicting suicidal behavior when stratifying by borderline personality disorder, and that should be viewed as the ideal target for further research on suicidal behavior and for the clinical assessment of suicide risk [12]. Conner et al. have concluded that both reactive aggression and proactive aggression is associated with suicidal behavior among substance-dependent patients [13]. Swogger et al's study has further suggested that aggression acts as an important mediator of the relationship between childhood physical abuse and suicide attempts among criminal offenders, supporting the importance of aggression treatment in suicide prevention programs. [14]. Furthermore, school-based studies have revealed: (1) that suicide-only adolescents have higher levels of overt and covert aggression than non-violent and non-suicide ones, and higher levels of covert aggression than violent-only ones; (2) that those who scored higher on reactive aggression had a greater risk for suicide behaviors than those with higher score on proactive aggression [15], [16]. Finally, as a behavioral marker of a high level of aggression, violent method accounts for the majority of suicides in the United States, especially firearms [17].

Additionally, evidence to date indicating the possibility that interventions directed at aggression may reduce the risk for suicidal behavior is numerous. For example, Lubell suggested a promising strategy of integrating suicide and violence prevention based on their shared influences to maximize the effectiveness and efficiency of prevention programs [18]. Other researchers also found that violent behavior in schools acts as a predictor of suicidal ideation, plans, and attempts among adolescents and stated the importance of combining violence and suicide prevention efforts [19], [20]. A Korean researcher tested the effectiveness of an integrated suicide-violence prevention program through a case-control study and found that this integrated program increased self-esteem and reduced aggression and suicidal behavior scores significantly [21].

On the basis of the above-mentioned studies, it must be concluded that the relationship between aggression/violence and suicidal behavior is without a doubt in the West. In China, however, there are no large studies investigating the effect of aggression on suicidal behavior. But we cannot replicate findings in the West directly to the Chinese population because suicidal behavior in China has its own characteristics due to different cultural, political, and social climates, and access to lethal methods for suicide and health care [4], [22], [23]. For example, the male to female gender ratio for suicide in China (1∶1.3) is much lower than that of western countries, where the typical male to female ratio is between 2∶1 and 4∶1 [24], [25]. Also, neither the main cause nor the most common method of suicide in China is consistent with those in most western countries (mental health disorders vs. family conflicts; violent methods like firearm vs. nonviolent methods like pesticide poisoning) [26], [27]. Discrimination against females, no religious or legal injunctions against suicide, the availability of suicide means (e.g. pesticides) and a longstanding tradition of using suicide as a fighting mean against parents or unfair things may partly explain those unique features [4], [28]. As a result, it is risky to apply the findings about the association between aggression and suicide behavior in western population directly to adolescents in China.

Moreover, Shaffer concluded that aggressive/violent outbursts and depression or withdrawal were two characteristics of people who were more vulnerable to suicide tendencies in 1970s [29]. According to this theory, most of suicide in the West is of the latter type since mental illness, substance use disorders and alcohol use disorders are factors that most consistently associated with suicide. In China, however, a recent study about the psychological strain theory of suicide among Chinese adolescents has formed a challenge to the psychiatric model that is well-established in the West [30]. Besides, Cui's study among Chinese adolescents has revealed that the special problems related to peer relationships, especially physical fighting and lack of peer association, were significantly related to suicide behavior [31]. A suicidal temperament/personality theory has suggested that impulsiveness, aggressiveness, anger, and hostility are crucial predispositions mediating suicidal behavior [32]–[34]. As a result, we need to study the relationship between aggression and suicide to better understand the risk factors for suicide and suicide behavior in China. And further investigations should be done to explore how trait aggression predicts suicidal behavior among Chinese adolescents.

The aim of the present study was to explore which forms of trait aggression were associated with suicidal behavior, as well as to reestimate the prevalence of suicide ideation, plans and attempts among adolescents of urban areas in China. . Based on literatures reviewed above, we hypothesized that physical aggression, anger, and hostility would be risk factors of adolescents' suicidal behavior after adjusting for various risk factors.

## Methods

### Sample and procedure

This was a nationwide survey of 7^th^ through 12^th^ grade public high school students conducted in 2010.A representative sample of urban high school students was generated using the stratified randomized cluster-sampling method [35]. First, from all cities meeting the set of urban criteria initiated in the 2000 Chinese Population Census (population density of at least 1,500/sq.km, the local government locating there, and being a contiguous built-up area), fifteen cities in five provinces (Guangdong, Anhui, Hubei, Heilongjiang, and Yunnan) were selected which were representative geographically, culturally and economically. [36]–[38].Second, from all public high schools in these cities, the initial sample of 82 schools was sampled systematically, including 40 junior high schools and 42 senior high schools. Among them, however, six senior high schools declined to have students participate. Finally, we had classes as the test unit and recruited a sample of 15,738 students for participation. Of those students, 14,537 returned their completed questionnaire, with a response rate of 92.4%. Accordingly, the final sample consisted of 7,249 junior (49.9%) and 7,288 senior (50.1%) high school students and had a mean age of 15.1 years (*SD* = 1.87, range = 10–18). Of them 7,485 (51.5%) were girls.

Data were collected via an 84-item self-reported questionnaire by a group of trained interviewers in classrooms during regular school hours to maximize student eligibility. Before completing the questionnaire, students were told to read the instruction carefully, which informed them that honest answers were preferred and their answers would be for scientific research only. About one class hour (nearly 45 minutes) were required to complete the questionnaire.

Our survey was approved by the Ethical Committee of Medical Association of Tongji Medical College of Huazhong University of Science and Technology and the Local Education Committee. In advance of the data collection, we got permission from all target schools who act in loco parentis in China, and also obtained consent from students who were invited to participate in the survey.

### Instrumentation

#### Suicidal behavior

Suicidal behavior was evaluated by four self-reported questions which had been found to be reliable sources of primary data about suicidality [7], [39]. They were “Have you ever thought about killing yourself during the past 12 months?”, “Have you ever made a specific plan about how you would kill yourself during the past 12 months?”, “How many times have you deliberately tried to kill yourself during the past 12 months?” and “If you attempted suicide during the past 12 months, what was the result of that attempt?” Participants were asked to answer the first three questions with “No” (scored 0) or “Yes” (scored 1). Suicide attempters needed to answer the last one with four options: be found and required medical care; be found and required no medical care; regretted and stop by yourself; unsuccessful due to other reasons. Depending on their answers, they would be considered categorically as suicide ideation, planner or attempter.

#### Trait aggression

The 34-item form of Buss and Warren's Aggression Questionnaire (BWAQ) was administered to assess trait aggression [40], [41]. Each item was answered on a 5-point Likert scale ranging from 1 (not at all like me) to 5 (completely like me). The questionnaire measured five constructs related to aggression: physical aggression (PHY), verbal aggression (VER), anger (ANG), hostility (HOS), and indirect aggression (IND). The Chinese version of BWAQ has been reported to have good psychometric properties (e.g. internal reliability: physical aggression = .81; verbal aggression = .71; anger = .64; hostility = .61; indirect aggression = .62) [42]. The overall Cronbach's α with the present sample was .87, and the internal consistency estimate for each subscale was .75, .51, .61, .69, and .58, respectively.

#### Self-esteem

The Chinese version of Rosenberg's Self-esteem Scale was used [43]. Each item was rated on a 4-point scale from “1 = not at all like me” to “4 = completely like me”. The internal consistency estimate in this study was .82.

#### Parent and peer attachment

The Inventory of Parent and Peer Attachment was used to measure the awareness of their attachment with parents and peers [44]. There are three subscales (mother attachment, father attachment, and peer attachment) and each of them consists of 25 items that are rated on a 5-point scale. Cronbach's α with the current sample was .91, .91, and .92, respectively.

#### Other risk factors

A wide range of social, family and school factors was included. They are city size (big/medium/small), perceived social atmosphere (good/fair/poor), family structure (extended or nuclear family/step family/single-parent family/grandparent family/others), one-child family (yes/no), accordance of parenting styles (yes/no), perceived family income (high/average/low), perceived school atmosphere (good/fair/poor), perceived relationship with teachers and classmates (good/fair/poor), academic performance (good/fair/poor), numbers of close friends (none/one and above), and satisfaction of appearance (satisfied/fair/unsatisfied).

### Statistical Analysis

According to Buss and Warren's interpretation of trait aggression, we grouped five continuous variables (composite score of physical aggression, verbal aggression, anger, hostility, and indirect aggression) into seven categories: very low, low, low average, average, high average, high, and very high. However, the cutpoints in this study were relatively lower than those Buss and Warren proposed in the BWAQ manual, because they originated from an Chinese norm that have been calculated based on the nationwide survey in 2010 [45]. The age ranging from 12 to 18 was viewed as a multinomial categorical variable.

Univariate logistic regression models were conducted then to explore the impact of various risk factors on suicidal behavior first. Then, after considering the sampling weight, a series of multivariate logistic regression analyses were finally performed to examine the magnitude and nature of association between trait aggression and suicide behavior after controlling for an arrary of risk factors. Model I, II, III tested the association between trait aggression and suicide ideation, plan, and attempt, respectively. Covariates of Model I, IIa, IIIa included gender, age, self-esteem, parent and peer attachment, and other factors that that were significant in univariate models. Model IIb was the one adjusting for suicide ideation based on Model IIa. Model IIIb was the one controlling for suicide ideation on the basis of Model IIIa, while Model IIIc was the one adjusting for suicide plan on the basis of Model IIIb. Before modeling, collinearity diagnostics were performed to test collinearity among model covariates.

All Odd Ratios (ORs) and Confidence interval (CI) of logistic regression models were presented. Students without suicidal behavior constituted the reference in all analyses. Wald statistics were used in all statistical tests of the regression estimates or ORs. Significance was set at the .05 level, and all tests were two-tailed. Additionally, for all regression analyses, the sampling weight, a product of reciprocal of the probability of multiple sampling, was taken into account, since the multistage cluster sampling were applied in this study. All statistical analyses were conducted in Statistical Package for Social Sciences software (SPSS for Windows 15.0, SPSS Inc., Chicago, IL).

## Results

Overall, 18.5% of the sample reported having thought about killing themselves during the past 12 months, 8.7% reported having seriously made plans to kill themselves, and 4.1% reported having attempted suicide. Among those attempters, 9.9% required medical care after being found; 13.7% required no medical care after being found and stopped; 34.2% regretted and stopped by themselves; and 42.2% failed due to other reasons. The gender ratios of suicide ideation, plans and attempts are 0.8∶1, 0.9∶1, and 1.1∶1 (males∶ females), respectively.

Table S1 shows results of descriptive analysis of demographic characteristics and various risk factors and ORs of those factors for predicting suicide ideation, plans and attempt in univariate logistic regression models. As shown in the table, a gender difference was found in suicidal ideation but not in suicide plans and attempts (female: 20.1%, 9.0%, and 3.9%; male: 16.8%, 8.4%, and 4.3%). The odds of suicide behavior among middle age group was 1.5 times to twice those among students aged 16 or above. Higher score in five subscales of trait aggression was significantly associated with elevated probability of suicide behavior (*p*<0.001).

Collinearity diagnostics reveal that no major collinearity problems among variables in each model are observed. The results of the multivariate models are shown in Table S2 and Table S3. From Table S2, the above average levels of HOS and the very high level of ANG were found to be positively associated with the odds of suicidal ideation after controlling for various risk factors (Model I). The above average levels of HOS and PHY had a significant positive relation with the odds of suicide plans even when suicide ideation was taken into account (Model IIa; Model IIb). In Model IIIa, high hostility and low anger were more likely to increase the odds of suicide attempts. After controlling for suicide ideation and plans, however, low anger remained a risk factor, but high hostility did not (Model IIIb; Model IIIc).

## Discussion

The main findings of this study enhance our understanding of suicide ideation, plans and attempts among Chinese adolescents. First, there is evidence of the association between trait aggression and suicide ideation, plans and attempts, indicating that hostility, physical aggression and trait anger predicted suicidal behavior among adolescents. Second, this study reported that approximately 18.5% of the school-based sample reported having suicide ideation, 8.7% reported having suicide plans, and 4.1% reported having suicide attempts during the past one year in China.

In a systematic review of adolescent suicidal phenomena, Evans et al. have reported that the average rate of adolescents reporting suicide thoughts in the past one year in the West was 19.3% (95% CI, 11.7–27.0), 12.4% (95% CI, 8.8–15.9) reporting suicide plans, and 6.4% (95% CI, 5.4–7.5) reporting suicide attempts [6]. In this study, however, these rates are a little lower than those findings. One of the reasons for these differences is that that we did not study rural areas, where risks for suicidal behavior are greater (suicide ideation: 19.3%, suicide plans: 10.5%, suicide attempts: 7.0%) [7]. As a result, our findings may underestimate the prevalence of suicide behaviors among adolescents in China as a whole. Second, differences in the age distribution of study samples may partly account for these differences. The mean age of this sample is a little smaller because of including more young children. Therefore, our estimates of suicide plans and attempts are not comparable to the data from the West. Third, Evans et al. have also found Asian adolescents have relatively lower rates of suicidal phenomena than other adolescents [6]. Our findings confirm this comment exactly. Besides, these differences are in line with the relatively lower suicide rates in 5–14 and 15–24 age groups in China than those in the West.

In this study, there is some evidence of nature and severity of the association between suicide behaviors and trait aggression, indicating the relative importance of different aggression forms in predicting suicidal behavior: (1) high hostility and trait anger were risk factors for suicide ideation when controlled for self-esteem, parent and peer attachment, demographic, family and school characteristics; (2) high hostility and physical aggression were risk factors for suicide plans after adjusted for other variables, and when current suicide ideation was controlled the association between high physical aggression remained significant; (3) high hostility and low trait anger were risk factors for suicide attempts among adolescents, and low trait anger remained to be a good predictor for suicide attempts when prior suicide ideation and plans were adjusted.

These findings are reasonable and imply that pathological mechanism of the three patterns of suicide behaviors might be somewhat distinctive, although suicidal behaviors overlap conceptually [46], [47]. First, according to the construction of BWAQ, hostility, the cognitive component of trait aggression, includes ‘feelings of ill will and injustice’ and anger, as the affective component, consists of ‘physiological arousal and preparation for aggression’ [40]. Our finding that hostility and trait anger can be used to predict suicide behavior confirms the validity of emotion-cognition model in predicting dangerous outcomes [48].

Second, since the BWAQ was developed from the Buss and Durke Hostility Inventory (BDHI), the hostility subscale still has the properties and constructs of its prototype. In BDHI, the neurotic hostility is not only associated with traits that predict distrust of others, vulnerability to stress, poor coping and frequent negative affect, but also influenced anxiety and depression [49]. And all the traits mentioned above are commonly known as risk factors accounting for youth suicide behaviors. Therefore, our finding that the hostility scale of the BWAQ, to great extent, predicted all categories of suicide behaviors supports the construct validity of the subscale of BWAQ.

Third, the aetiological profiles of suicidal behavior are quite similar, but not identical. Some researchers have argued that the distinctive part of the three categories of suicidal behavior probably rests on degree of the same risk factors [50]. On the contrary, other researchers hold a different view that their differences in aetiology were resulted from different risk factors related to suicidal behavior [51]. Actually, the two views are not contradictive, but relatively complementary. The current study confirmed the point. For example, trait anger was the common predictor for both suicide ideation and attempts, but the nature was quite different. High physical aggression was a good predictor only for suicide plans. Finally, with regard to the predictive function of low trait anger for suicide attempts, we speculate that it might be explained by serious depressive symptoms of attempter. We could not examine this here since depression was not included in this study. Additional research should be done to confirm these results.

This study may provide some important implications for understanding suicide behavior and preventing suicide in school environments. First, according to our findings, the risk of youth suicide behavior raised with the elevated severity of hostility and physical aggression, suicide prevention programs targeting at attenuating these traits may potentially be very impactful. Second, this research also underlines the need of close observation of students with low trait anger or being too silent or extremely obedient. This poses a challenge over the cultural tradition in China that obedience is one of the most important criteria of a good child.

Our findings must be viewed with caution given the study limitations. First, we are not able to directly determine the seriousness of the reported suicide attempts in this study, in terms of lethality and intent. It is probable that many youths who report attempts did not really intend to die. This may greatly inflate the rate for attempts. On the other hand, since we did not the rural aspect in the sample design, the rates for China reported in this study might be underestimated. Second, given the role of depression on aggressive youths, the lack of data on depression in the present study may somewhat attenuate our results. Third, the data were derived from a self-reported survey. Thus, these results are liable to all of the self-report biases, including underreporting and autobiographically memory errors of items in questionnaires [52]. Furthermore, although we employed standardized measures and procedures to maximize the representativeness of our sample, it cannot be denied that some sampling biases may limit the extent to which our findings are generalized to all students in urban areas of China. Fourth, this study is a cross-sectional study, which eliminates the causal efficacy of all data; therefore, we can only infer the direction of association between trait aggression and suicidal behavior. Further research, for example, prospective and longitudinal studies, should be done to validate or refute our findings. Finally, we chose forward method based on conditional parameter estimation as our analysis strategy. Nevertheless, it is undeniable that different analysis strategies we used may lead to different conclusions. Therefore, the models of our study many also be influenced by statistical approaches that could have been taken.

## Supporting Information

Table S1 **Descriptive analyses of demographic characteristics and various risk factors, and odds ratios of those factors for predicting suicide ideation, plans and attempts in univariate logistic regression models.**
(DOC)

Table S2 **Odds ratios of all subscales of trait aggression for predicting suicide ideation and plans after adjusted for sociodemographic and various risk factors in this sample.**
(DOC)

Table S3 **Odds ratios of all subscales of trait aggression for predicting suicide attempts after adjusted for sociodemographic and various risk factors in this sample.**
(DOC)
