# Supplementary material for: Hostility, Physical Aggression and Trait Anger as Predictors for Suicidal Behavior in Chinese Adolescents: A School-Based Study
Source: PLoS One. 2012 Feb 16;7(2):e31044. doi: 10.1371/journal.pone.0031044 (PMC3281042; doi:10.1371/journal.pone.0031044)
Supplement: Table S1 — Descriptive analyses of demographic characteristics and various risk factors, and odds ratios of those factors for predicting suicide ideation, plans and attempts in univariate logistic regression models. (DOC) [file pone.0031044.s001.doc]

Table S1 Descriptive analyses of demographic characteristics and various risk factors, and odds ratios of those factors for predicting suicide ideation, plans and attempts in univariate logistic regression models

| Characteristics | %/Mean±S.D. | Suicide ideation | |  | Suicide plans | |  | Suicide attempts | |
| --- | --- | --- | --- | --- | --- | --- | --- | --- | --- |
|  |  | ORs | 95% CI |  | ORs | 95% CI |  | ORs | 95% CI |
| Gender (Female) | 51.5 | 1.24*** | 1.14-1.34 |  | 1.07 | 0.96-1.21 |  | 1.12 | 0.95-1.32 |
| Age |  |  |  |  |  |  |  |  |  |
| 12 or less | 9.6 | 1.08 | 0.92-1.26 |  | 1.38** | 1.11-1.71 |  | 1.48*** | 1.24-1.77 |
| 13-15 | 47.2 | 1.33*** | 1.22-1.45 |  | 1.71*** | 1.51-1.94 |  | 1.61** | 1.21-2.13 |
| 16+ | 43.2 | 1.00 |  |  | 1.00 |  |  | 1.00 |  |
| City size |  |  |  |  |  |  |  |  |  |
| Big | 34.4 | 1.39*** | 1.25-1.54 |  | 1.27** | 1.10-1.47 |  | 1.44** | 1.17-1.78 |
| Medium | 32.4 | 1.19** | 1.07-1.33 |  | 1.00 | 0.86-1.16 |  | 1.16 | 0.93-1.45 |
| Small | 33.2 | 1.00 |  |  | 1.00 |  |  | 1.00 |  |
| Social atmosphere^a^ |  |  |  |  |  |  |  |  |  |
| Good | 48.0 | 1.00 |  |  | 1.00 |  |  | 1.00 |  |
| Fair | 47.5 | 1.38*** | 1.26-1.51 |  | 1.39*** | 1.23-1.58 |  | 1.24* | 1.04-1.48 |
| Poor | 4.4 | 2.61*** | 2.19-3.12 |  | 2.84*** | 2.28-3.55 |  | 3.12*** | 2.34-4.16 |
| Family structure |  |  |  |  |  |  |  |  |  |
| Extended or nuclear family^b^ | 91.3 | 1.00 |  |  | 1.00 |  |  | 1.00 |  |
| Step family^c^ | 2.3 | 1.42** | 1.10-1.83 |  | 1.92*** | 1.41-2.61 |  | 2.26*** | 1.52-3.36 |
| Single-parent family^d^ | 4.5 | 1.39*** | 1.16-1.68 |  | 1.46** | 1.15-1.87 |  | 1.42* | 1.00-2.00 |
| Grandparent family^e^ | 1.6 | 1.30 | 0.95-1.79 |  | 1.65* | 1.11-2.44 |  | 1.03 | 0.52-2.01 |
| Others | 0.4 | 2.03* | 1.14-3.60 |  | 2.77** | 1.43-5.37 |  | 1.41 | 0.44-4.54 |
| One-child family (Yes) | 55.0 | 1.22*** | 1.12-1.33 |  | 1.24*** | 1.10-1.40 |  | 1.29** | 1.09-1.53 |
| Accordance of parenting styles ^f^ (No) | 21.6 | 1.92*** | 1.75-2.11 |  | 2.20*** | 1.94-2.49 |  | 2.11*** | 1.77-2.51 |
| Family income |  |  |  |  |  |  |  |  |  |
| High | 14.1 | 1.47*** | 1.28-1.69 |  | 1.46*** | 1.21-1.77 |  | 1.73*** | 1.32-2.27 |
| Average | 61.6 | 1.31*** | 1.17-1.45 |  | 1.29** | 1.11-1.50 |  | 1.36** | 1.09-1.68 |
| Low | 24.4 | 1.00 |  |  | 1.00 |  |  | 1.00 |  |
| School atmosphere^g^ |  |  |  |  |  |  |  |  |  |
| Good | 45.2 | 1.00 |  |  | 1.00 |  |  | 1.00 |  |
| Fair | 48.5 | 1.59*** | 1.45-1.75 |  | 1.66*** | 1.45-1.89 |  | 1.65*** | 1.36-2.00 |
| Poor | 6.3 | 2.46*** | 2.08-2.89 |  | 3.27*** | 2.66-4.01 |  | 3.92*** | 2.99-5.13 |

（*Continued*）

*Table S1 Continued*

| Characteristics | %/Mean±S.D. | Suicide ideation | |  | Suicide plans | |  | Suicide attempts | |
| --- | --- | --- | --- | --- | --- | --- | --- | --- | --- |
|  |  | ORs | 95% CI |  | ORs | 95% CI |  | ORs | 95% CI |
| Relationship with teachers |  |  |  |  |  |  |  |  |  |
| Good | 51.4 | 1.00 |  |  | 1.00 |  |  | 1.00 |  |
| Fair | 46.5 | 1.53*** | 1.40-1.67 |  | 1.47*** | 1.31-1.66 |  | 1.53*** | 1.29-1.82 |
| Poor | 2.1 | 3.90*** | 3.08-4.93 |  | 4.66*** | 3.56-6.10 |  | 5.84*** | 4.19-8.14 |
| Relationship with classmates |  |  |  |  |  |  |  |  |  |
| Good | 71.2 | 1.00 |  |  | 1.00 |  |  | 1.00 |  |
| Fair | 27.6 | 1.50*** | 1.37-1.65 |  | 1.57*** | 1.39-1.77 |  | 1.53*** | 1.28-1.82 |
| Poor | 1.2 | 2.66*** | 1.95-3.61 |  | 3.29*** | 2.29-4.72 |  | 3.51*** | 2.20-5.60 |
| Academic performance |  |  |  |  |  |  |  |  |  |
| Good | 38.6 | 1.00 |  |  | 1.00 |  |  | 1.00 |  |
| Fair | 34.0 | 1.12* | 1.01-1.24 |  | 1.06 | 0.92-1.23 |  | 1.28* | 1.04-1.58 |
| Poor | 27.4 | 1.56*** | 1.40-1.72 |  | 1.60*** | 1.39-1.84 |  | 1.93*** | 1.58-2.36 |
| Number of friends (None) | 1.2 | 3.03*** | 2.25-4.08 |  | 2.99*** | 2.09-4.26 |  | 4.74*** | 3.18-7.08 |
| Satisfaction of appearance |  |  |  |  |  |  |  |  |  |
| Satisfied | 49.5 | 1.00 |  |  | 1.00 |  |  | 1.00 |  |
| Fair | 45.3 | 1.45*** | 1.32-1.58 |  | 1.33*** | 1.17-1.50 |  | 1.35** | 1.13-1.60 |
| Unsatisfied | 5.3 | 3.12*** | 2.66-3.66 |  | 3.78*** | 3.12-4.58 |  | 2.89*** | 2.19-3.81 |
| Trait aggression |  |  |  |  |  |  |  |  |  |
| PHY | 13.38±4.894 | 1.08*** | 1.07-1.09 |  | 1.09*** | 1.08-1.10 |  | 1.10*** | 1.08-1.11 |
| VER | 12.95±3.324 | 1.07*** | 1.06-1.08 |  | 1.09*** | 1.07-1.11 |  | 1.05*** | 1.03-1.08 |
| IND | 12.49±3.897 | 1.12*** | 1.11-1.13 |  | 1.13*** | 1.11-1.14 |  | 1.12*** | 1.10-1.14 |
| ANG | 15.90±4.649 | 1.10*** | 1.09-1.11 |  | 1.11*** | 1.10-1.13 |  | 1.12*** | 1.10-1.13 |
| HOS | 17.31±5.182 | 1.14*** | 1.13-1.15 |  | 1.15*** | 1.14-1.17 |  | 1.13*** | 1.12-1.15 |
| Parental and peer attachment |  |  |  |  |  |  |  |  |  |
| Mother attachment | 87.80±16.165 | 0.97*** | 0.96-0.97 |  | 0.96*** | 0.95-0.96 |  | 0.95*** | 0.95-0.96 |
| Father attachment | 85.45±17.317 | 0.97*** | 0.97-0.98 |  | 0.96*** | 0.96-0.97 |  | 0.96*** | 0.96-0.97 |
| Peer attachment | 93.41±15.585 | 0.99*** | 0.98-0.99 |  | 0.99*** | 0.98-0.99 |  | 0.99*** | 0.98-0.99 |
| Self-esteem | 29.94±5.016 | 0.91*** | 0.90-0.92 |  | 0.89*** | 0.87-0.90 |  | 0.87*** | 0.85-0.88 |
| Suicide ideation (Yes/ No) | 18.5 |  |  |  | 117.34*** | 92.86-148.27 |  | 47.15*** | 38.10-61.58 |
| Suicide plan (Yes/ No) | 8.7 |  |  |  |  |  |  | 31.13*** | 25.90-37.43 |
| Suicide attempts (Yes/ No) | 4.1 |  |  |  |  |  |  |  |  |

Note: a. Social atmosphere: means a set of manners, morals, and behavior of people in various aspects of social life; being evaluated by “good”, “fair”, or “poor”; b. Extended or nuclear family: living with both father and mother; c. Step family: living with stepfather or stepmother; d. Single-parent family: living with father or mother; e. grandparent family: living with grandparents.

f. accordance of parenting styles: whether the parenting style of father is the same as that of mother in a family; g. School atmosphere: means a set of manners, morals, and behavior of students in various aspects of school life. * *p*＜0.05; ** *p*＜0.01; *** *p*＜0.001
