# Supplementary material for: Hostility, Physical Aggression and Trait Anger as Predictors for Suicidal Behavior in Chinese Adolescents: A School-Based Study
Source: PLoS One. 2012 Feb 16;7(2):e31044. doi: 10.1371/journal.pone.0031044 (PMC3281042; doi:10.1371/journal.pone.0031044)
Supplement: Table S2 — Odds ratios of all subscales of trait aggression for predicting suicide ideation and plans after adjusted for sociodemographic and various risk factors in this sample. (DOC) [file pone.0031044.s002.doc]

Table S2 Odds ratios of all subscales of trait aggression for predicting suicide ideation and plans after adjusted for sociodemographic and various risk factors in this sample

|  | Model Ⅰ | |  | Model Ⅱa | |  | Model Ⅱb | |
| --- | --- | --- | --- | --- | --- | --- | --- | --- |
|  | ORs | 95% CI |  | ORs | 95% CI |  | ORs | 95% CI |
| PHY Low |  |  |  | 1.00 |  |  | 1.00 |  |
| Low average |  |  |  | 1.04 | 0.68-1.60 |  | 1.01 | 0.65-1.58 |
| Average |  |  |  | 1.52** | 1.12-2.07 |  | 1.37 | 0.99-1.90 |
| High average |  |  |  | 1.68** | 1.20-2.36 |  | 1.45* | 1.01-2.08 |
| High |  |  |  | 1.79** | 1.27-2.54 |  | 1.75** | 1.21-2.54 |
| Very high |  |  |  | 2.09** | 1.29-3.39 |  | 1.44 | 0.84-2.45 |
| ANG Very low | 1.00 |  |  |  |  |  |  |  |
| Low | 1.50 | 0.91-2.46 |  |  |  |  |  |  |
| Low average | 1.08 | 0.67-1.74 |  |  |  |  |  |  |
| Average | 1.28 | 0.81-2.01 |  |  |  |  |  |  |
| High average | 1.25 | 0.78-2.00 |  |  |  |  |  |  |
| High | 1.49 | 0.93-2.38 |  |  |  |  |  |  |
| Very high | 2.12** | 1.24-3.65 |  |  |  |  |  |  |
| HOS Very low | 1.00 |  |  |  |  |  |  |  |
| Low | 1.12 | 0.51-2.47 |  | 1.00 |  |  | 1.00 |  |
| Low average | 1.59 | 0.74-3.42 |  | 1.11 | 0.63-1.96 |  | 0.93 | 0.50-1.75 |
| Average | 2.63** | 1.24-5.57 |  | 2.37*** | 1.50-3.75 |  | 1.41 | 0.85-2.36 |
| High average | 3.33*** | 1.55-7.13 |  | 3.67*** | 2.28-5.90 |  | 2.04** | 1.19-3.47 |
| High | 4.30*** | 2.00-9.23 |  | 5.14*** | 3.20-8.28 |  | 2.51** | 1.48-4.26 |
| Very high | 7.18*** | 3.18-16.18 |  | 10.50*** | 6.08-18.13 |  | 4.68*** | 2.50-8.77 |
| Suicide ideation (Yes) |  |  |  |  |  |  | 86.69*** | 64.08-117.26 |
| Gender | 1.19** | 1.06-1.34 |  |  |  |  | 0.79** | 0.64-0.94 |
| Age | 0.83*** | 0.75-0.91 |  | 0.69*** | 0.61-0.78 |  | 0.72*** | 0.61-0.83 |
| City size | 0.89** | 0.83-0.96 |  |  |  |  |  |  |
| One-child family (Yes) | 1.18** | 1.04-1.32 |  |  |  |  |  |  |
| Accordance of parenting styles (No) | 1.28*** | 1.13-1.46 |  | 1.49*** | 1.26-1.75 |  | 1.37** | 1.12-1.68 |
| School atmosphere | 1.23*** | 1.12-1.35 |  | 1.26*** | 1.11-1.43 |  |  |  |
| Academic performance |  |  |  |  |  |  |  |  |
| Number of friends | 1.84** | 1.23-2.75 |  | 1.77* | 1.11-2.82 |  |  |  |
| Satisfaction of appearance |  |  |  | 0.87* | 0.76-0.99 |  | 0.85* | 0.73-0.99 |
| Mother attachment | 0.98*** | 0.98-0.99 |  | 0.98*** | 0.98-0.99 |  | 0.99* | 0.99-1.00 |
| Father attachment | 0.99*** | 0.98-0.99 |  | 0.99*** | 0.98-0.99 |  | 0.99* | 0.99-1.00 |
| Peer attachment | 1.01*** | 1.01-1.02 |  | 1.01*** | 1.01-1.02 |  |  |  |
| Self-esteem | 0.94*** | 0.92-0.95 |  | 0.94*** | 0.93-0.96 |  |  |  |
| Constant | 7.06 |  |  | 3.91 |  |  | 0.08 |  |

Note: PHY=Physical aggression; VER=Verbal aggression; ANG=Anger; HOS=Hostility; IND=Indirect aggression. * *p*＜0.05; ** *p*＜0.01; *** *p*＜0.001
