# Supplementary material for: Hostility, Physical Aggression and Trait Anger as Predictors for Suicidal Behavior in Chinese Adolescents: A School-Based Study
Source: PLoS One. 2012 Feb 16;7(2):e31044. doi: 10.1371/journal.pone.0031044 (PMC3281042; doi:10.1371/journal.pone.0031044)
Supplement: Table S3 — Odds ratios of all subscales of trait aggression for predicting suicide attempts after adjusted for sociodemographic and various risk factors in this sample. (DOC) [file pone.0031044.s003.doc]

Table S3 Odds ratios of all subscales of trait aggression for predicting suicide attempts after adjusted for sociodemographic and various risk factors in this sample

|  | Model Ⅲa | |  | Model Ⅲb | |  | Model Ⅲc | |
| --- | --- | --- | --- | --- | --- | --- | --- | --- |
|  | ORs | 95% CI |  | ORs | 95% CI |  | ORs | 95% CI |
| ANG Very low | 1.00 |  |  | 1.00 |  |  | 1.00 |  |
| Low | 0.41* | 0.19-0.87 |  | 0.19*** | 0.08-0.43 |  | 0.19*** | 0.08-0.44 |
| Low average | 0.28** | 0.14-0.58 |  | 0.17*** | 0.08-0.37 |  | 0.18*** | 0.08-0.39 |
| Average | 0.43** | 0.23-0.81 |  | 0.23*** | 0.11-0.47 |  | 0.22*** | 0.11-0.44 |
| High average | 0.39** | 0.20-0.74 |  | 0.22*** | 0.10-0.46 |  | 0.21*** | 0.10-0.44 |
| High | 0.61 | 0.32-1.16 |  | 0.31** | 0.15-0.64 |  | 0.31** | 0.15-0.64 |
| Very high | 0.54 | 0.25-1.18 |  | 0.23** | 0.10-0.55 |  | 0.24* | 0.10-0.56 |
| HOS Very low |  |  |  |  |  |  |  |  |
| Low | 1.00 |  |  |  |  |  |  |  |
| Low average | 2.67* | 1.20-5.96 |  |  |  |  |  |  |
| Average | 3.04** | 1.47-6.30 |  |  |  |  |  |  |
| High average | 3.38** | 1.57-7.26 |  |  |  |  |  |  |
| High | 5.56*** | 2.61-11.88 |  |  |  |  |  |  |
| Very high | 8.83*** | 3.81-20.48 |  |  |  |  |  |  |
| Suicide ideation (Yes) |  |  |  | 39.64*** | 27.47-57.19 |  | 19.73*** | 13.23-29.42 |
| Suicide plan (Yes) |  |  |  |  |  |  | 3.96*** | 3.03-5.17 |
| Age | 0.64*** | 0.54-0.76 |  | 0.67*** | 0.56-0.82 |  | 0.72** | 0.59-0.88 |
| City size | 0.82** | 0.71-0.94 |  |  |  |  |  |  |
| School atmosphere | 1.42*** | 1.17-1.72 |  | 1.36** | 1.12-1.64 |  | 1.34** | 1.10-1.63 |
| Relationship with teachers | 1.26* | 1.01-1.56 |  |  |  |  |  |  |
| Academic performance | 1.18* | 1.03-1.37 |  | 1.24** | 1.08-1.43 |  | 1.23** | 1.07-1.43 |
| Number of friends | 2.81*** | 1.65-4.80 |  | 2.29** | 1.30-4.04 |  | 2.37** | 1.33-4.22 |
| Mother attachment | 0.98*** | 0.97-0.99 |  | 0.99*** | 0.98-0.99 |  | 0.99** | 0.98-1.00 |
| Father attachment | 0.99* | 0.99-1.00 |  |  |  |  |  |  |
| Peer attachment | 1.01** | 1.01-1.02 |  |  |  |  |  |  |
| Self-esteem | 0.95*** | 0.93-0.97 |  |  |  |  |  |  |
| Constant | 0.75 |  |  | 0.02 |  |  | 0.01 |  |

Note: PHY=Physical aggression; VER=Verbal aggression; ANG=Anger; HOS=Hostility; IND=Indirect aggression. * *p*＜0.05; ** *p*＜0.01; *** *p*＜0.001
